# Assessing observational studies of medical treatments

**DOI:** 10.1186/1742-7622-2-8

**Published:** 2005-09-01

**Authors:** Arthur Hartz, Suzanne Bentler, Mary Charlton, Douglas Lanska, Yogita Butani, G Mustafa Soomro, Kjell Benson

**Affiliations:** 1 University of Iowa, College of Medicine, Department of Family Medicine, Iowa City, IA 52242 USA; 2 University of Iowa, College of Medicine, Department of Family Medicine, Iowa City, IA 52242 USA; 3 University of Iowa, College of Medicine, Department of Family Medicine, Iowa City, IA 52242 USA; 4 VA Medical Center, 500 East Veterans Street, Tomah, WI 54660 USA; 5 University of Iowa, College of Medicine, Department of Family Medicine, Iowa City, IA 52242 USA; 6 Section of Community Psychiatry, St. George's Hospital Medical School, London, UK; 7 Family Practice Clinic, North Colorado Medical Center, Greeley, Colorado USA

**Keywords:** comparative studies, epidemiologic research design, meta-analysis, reproducibility of results, reporting criteria for observational studies

## Abstract

**Background:**

Previous studies have assessed the validity of the observational study design by comparing results of studies using this design to results from randomized controlled trials. The present study examined design features of observational studies that could have influenced these comparisons.

**Methods:**

To find at least 4 observational studies that evaluated the same treatment, we reviewed meta-analyses comparing observational studies and randomized controlled trials for the assessment of medical treatments. Details critical for interpretation of these studies were abstracted and analyzed qualitatively.

**Results:**

Individual articles reviewed included 61 observational studies that assessed 10 treatment comparisons evaluated in two studies comparing randomized controlled trials and observational studies. The majority of studies did not report the following information: details of primary and ancillary treatments, outcome definitions, length of follow-up, inclusion/exclusion criteria, patient characteristics relevant to prognosis or treatment response, or assessment of possible confounding. When information was reported, variations in treatment specifics, outcome definition or confounding were identified as possible causes of differences between observational studies and randomized controlled trials, and of heterogeneity in observational studies.

**Conclusion:**

Reporting of observational studies of medical treatments was often inadequate to compare study designs or allow other meaningful interpretation of results. All observational studies should report details of treatment, outcome assessment, patient characteristics, and confounding assessment.

## Introduction

As compared to randomized controlled trials of medical interventions, observational studies (OSs) are likely to be more timely, less expensive, and include patients more representative of usual clinical practice. In addition, OSs avoid ethical issues caused by compromising the patients' or physicians' therapeutic choices. However, their validity is vigorously debated [1,2]. Concerns about validity were heightened by a well-publicized randomized controlled trial (RCT) that found an increased risk of heart disease for women on hormone replacement therapy [3]. This study contradicted results from several previous high profile OSs [4-6].

It is possible that OSs are particularly ill-suited to evaluate hormone replacement therapy. The choice of this therapy is greatly influenced by patient ideas about youth and femininity, which may be highly confounded by unmeasured factors affecting health. Not all OSs found a decreased risk, however [7,8], and one study found evidence that adjusting results for socioeconomic status yielded similar results to randomized controlled trials [9]. Other factors contributing to differences between the types of studies may include design features that do not necessarily influence validity, such as the older age of women in the randomized trials.

Even if OSs of hormone replacement therapy are shown to be invalid, the observational study design may still have a role in the assessment of other medical treatments. For example, these studies may give good results for evaluation of a surgical procedure that is determined primarily by physician familiarity with a specific treatment or by treatment availability. Support for the validity of some OSs comes from reviews that found that observational and randomized studies often give similar results [10-13].

The present study investigated the comparisons of OSs and RCTs in more depth. In addition to the comparisons, we examined design features of the OSs that could have influenced these comparisons. In the process of this examination, we assessed how well OSs of medical treatments were reported.

## Materials and methods

Each of the reviewed studies compared outcomes of a given medical treatment to outcomes of a comparison group, which was most often standard therapy. Studies were selected from articles that compared results from observational and randomized studies previously assessed in meta-analyses or systematic reviews [13,14]. The reason for including only studies previously included in systematic reviews or meta-analyses was to increase the likelihood that all articles on a given topic were reviewed. Meta-analyses of fewer than four OSs were excluded from our review, because they provided limited ability to evaluate influences of study design characteristics on results of OSs.

### Study characteristics abstracted

To determine characteristics that should be abstracted, we reviewed the literature as to how RCTs should be reported [15,16] and evaluated [17], how OSs should be reported and evaluated [18], and how patient and treatment characteristics influence RCTs [19]. Some information from RCTs was not relevant for OSs (e.g., blinding of the randomization process), some was relevant but not available in OSs (e.g., protocols for administering the primary treatment and managing intermediate outcomes), and some was not relevant to comparing studies (e.g., power of the study when data have already been collected).

Based on previous literature and our own experience with OSs, we developed the schema in Table 1 for study characteristics that could influence results by influencing either the study's applicability or validity. Factors that could influence applicability include specific characteristics of the treatments, outcomes, or subjects; results that apply to studies using specific types of treatments, outcome measurements or subjects may be valid, but may not be reproduced by other studies using different types of treatments, outcome measurements or subjects.

**Table 1:** Study Characteristics That Influence Results

**Characteristics That Influence Applicability**
**Treatment specifics**
Details of procedure
Details of ancillary treatments or management of intermediate outcomes
**Outcome**
Definition
Method of patient contact and assessment at follow-up
Length of follow-up
**Patient characteristics**
Study setting
Inclusion/exclusion criteria
Reported characteristics

**Characteristics That Influence Validity**
**Information bias**
Incorrectly ascertaining treatment or outcome
**Selection bias**
Pretreatment – subjects on different treatments have different risks
Post treatment – lost to follow-up depends on outcome and treatment
**Confounding**
Caused by pretreatment selection bias
Demonstrated by differences in risk factors between treatment groups
Possibly reduced by risk-adjustment

Factors that could influence validity include those that could contribute to confounding, selection, or information (also called measurement) bias [20]. Confounding arises when subjects who receiving one treatment differ in risk from subjects receiving another, independent of the effect of treatment. Selection bias occurs when the association between exposure and disease differs between those who complete a study and those in the target population. In cohort studies of medical treatments, such as those reviewed below, pre-treatment selection bias leads to confounding and post-treatment selection bias results from incomplete follow-up that differs according to both outcome and treatment. Information bias occurs when errors are made in assessing which treatment or outcome a patient had. Although post-treatment selection bias and information bias distort estimates of effect size, they were difficult to assess in the papers reviewed and were not recorded in our analysis.

This schema guided the type of information abstracted from the reviewed articles. Although it does not include all 27 items considered important for measuring the quality of OSs in one schema [18], it is conceptually simple and should include most study aspects that influence interpretation of results. For each article reviewed, we noted critical data elements omitted from the article.

We deemed that the likelihood of confounding would be increased if treatment choice were related to time, so that recent patients generally received one treatment, whereas patients from several years previously received another. Confounding could also be more likely if treatment was allocated on the basis of patient characteristics that contribute to treatment failure, either by the physician or through patient self-selection. Confounding was considered less likely if the physicians treating the patients used only one procedure. An implicit assumption in this criterion is that patient risk and quality of care are similar across physicians; this assumption may not be valid in all cases, but we wanted to judge the studies as generously as possible so that reports of deficiencies in these studies would be conservative. Another criterion for decreased likelihood of confounding was an abrupt change in patient care, so that all patients received one treatment before the change and all patients received another treatment after the change.

All data abstraction was from the original articles. Although the majority of articles were assessed independently by two different reviewers, some articles were only assessed by the same reviewer several months apart. Disagreements between reviewers, between reviews at different times, or between our reviewers and the published meta-analysis were resolved through discussion.

### Statistical methods

Results were reported as statistically significant if p < 0.05, although p-values were often much lower. We used a 2-by-2 χ^2^-test for contingency tables to compare OS and RCT subjects for pooled failure rates on the same treatment. Significantly different failure rates for OS and RCT studies of one treatment in a comparison but not the other is a sensitive indication of possible confounding in the OSs. Significantly different failure rates for both treatments suggest that the two types of studies may differ with respect to features that influence failure rates (e.g., patients, outcome measures, specifics of the treatment, or uses of ancillary treatments). We also evaluated whether it might be worthwhile to search for important study factors that caused heterogeneity by examining variation among OSs for failure rates of a given treatment. The p-value for the statistical significance of this variation was determined using a 2-by-k χ^2^-test for contingency tables, where k was the number of studies that evaluated a given treatment. Pearson's correlation coefficient, denoted r, was used to compute the p-value for the association between the failure rates in the treatment group and the failure rates in the control group at the 0.05 significance level.

Statistical methods were used to combine odds ratios from several studies and to test the difference between the summary odds ratios from the observational and randomized studies. To combine odds ratios from several studies and to find the standard error of the combined odds ratio, we used a fixed-effects calculation [21]. By using fixed- rather than random-effects calculations [22], we obtained smaller standard errors and decreased the chances of missing true differences. However, this method may increase the likelihood of finding spurious differences.

We tested the difference between two odds ratios using the equation

Z = (Ln_1 _- Ln_2_) / √(SE_1_^2 ^+ SE_2_^2^)

where Z has a normal distribution with mean zero and variance 1, Ln_1 _and Ln_2 _are the logarithms of the two odds ratios, and SE_1 _and SE_2 _are the standard errors of these logarithms. Heterogeneity in odds ratios was tested with the Breslow-Day test for homogeneity at the 0.05 significance level.

## Results

### Meta-analyses selected for review

The selected analyses are shown in Table 2. These analyses addressed 10 topics: anticoagulants for treatment of myocardial infarction, quinidine for atrial fibrillation, trial of labor for patients with a breech delivery, colposuspension compared to anterior colporrhaphy for urinary incontinence, colposuspension compared to needle suspension for urinary incontinence, transcutaneous electrical nerve stimulation (TENS) for treatment of postsurgical pain, early discharge following childbirth, hip screws for hip fracture, local anesthesia for patients with carotid endarterectomy, and hysterosalpingography (HSG) media on pregnancy.

**Table 2:** Meta-analyses Selected for Review

Brief Title	Year	Medical Condition	Treatment 1 v Treatment 2	Failure Outcome	No. of studies (RCT, OS)	Reasons for excluding OSs previously compared to RCTs [13]
Anticoagulants	1977	Myocardial Infarction	Control v Anticoagulants	Mortality	Ioannidis: (6, 12) Kunz: (6, 12)	Three used alternately assigned controls [23-25].
Quinidine	1992	Atrial fibrillation	Control v Quinidine	Relapse into atrial fibrillation	Ioannidis: (6, 5) Kunz: (6, 6)	One did not have 3-month follow-up [53].
Trial of Labor	1995	Breech delivery	No trial v Trial of Labor	5 minute Apgar	Ioannidis: (2, 6)	None excluded.
Colposuspension 1	1996	Incontinence	Anterior colporrhaphy v Colposuspension	No cure of incontinence	Ioannidis: (4, 11)	Five were in a foreign language [54-58].
Colposuspension 2	1996	Incontinence	Needle suspension v Colposuspension	No cure of incontinence	Ioannidis: (3, 9)	One used failed surgery instead of incontinence as an outcome [59]. One was a controlled trial [26]. Three were in a foreign language [55, 56, 60].
Transcutaneous Electrical Nerve Stimulation (TENS)	1996	Post-operative pain	Control v TENS	No pain relief	Ioannidis: (2, 4) Kunz: (17, 19)	None excluded.
Early Discharge	1997	Childbirth	Conventional v Early	Maternal Morbidity	Ioannidis: (1, 3)	Added one study from original meta-analysis [36].
Hip screw	1999	Hip fracture	Fixed nail plates v Sliding hip screw	Total complications	Ioannidis: (1, 6)	One "OS" was an RCT [27].
Local Anesthesia	1996	Carotid Endarterectomy	General v Local Anesthesia	Stroke or death	Ioannidis: (3, 14)	One from a non-peer reviewed abstract [61], 1 from unpublished data [62], one in a foreign language [63].
Hysterosalpingo-graphy (HSG)	1999	Infertility	Water v Oil in Hysterosalpingography	No Pregnancy	Ioannidis: (5, 6)	None excluded.

RCT = Randomized Controlled Trial

OS = Observational Study

With five exceptions, we considered as observational all studies that were considered observational in the reviewed meta-analyses [13]: three of these were excluded because they used alternately assigned controls [23-25], and two were RCTs [26,27]. We did not exclude studies that used historical controls. Seven additional studies that were not in English were excluded because we were not able to accurately abstract detailed information about them.

Some studies assessed more than one outcome. With one exception, we reported results for the same outcomes that were assessed in the study by Ioannidis et al [13]. The exception was the meta-analysis of quinidine [28]. The outcome used by Ioannidis et al. from that analysis was mortality, which was zero or near zero for most studies. We used relapse of atrial fibrillation following cardioversion, which was used by our other source of meta-analyses [14]. Failure rates were used to compute odds ratios not computed by the original studies. For some studies the success rates and odds ratios in the primary studies [29-32] differed from those reported by Ioannidis et al. [13] or the meta-analysis [28]. When there was a discrepancy, we used the rates reported in the primary studies. Rates in primary studies for positive endpoints (e.g. pregnancy) were converted to failure rates (e.g. no pregnancy).

### Comparisons of observational and randomized studies

**Figure 1:**
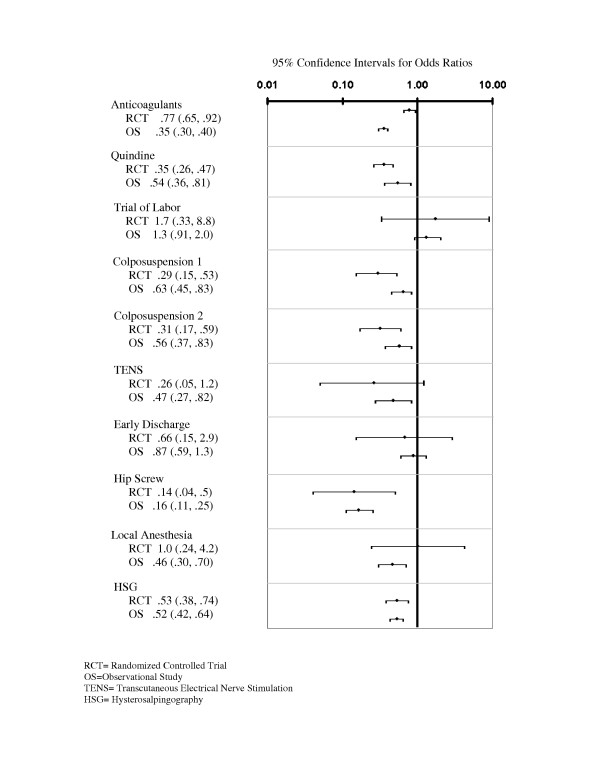
Comparison of Confidence Intervals for Combined Odds Ratios from Observational Studies and Randomized Controlled Trials.

The comparison of the combined odds ratios for the two types of studies are shown in Figure 1. In general, the confidence intervals were wider for the RCTs than for the OSs, reflecting the larger sample sizes for the OSs. Wide confidence intervals for randomized controlled studies of trial of labor, transcutaneous electrical nerve stimulation (TENS), early discharge, and local anesthesia prevented meaningful comparisons for these treatment areas. The only treatment area for which the odds ratios differed significantly was studies of anticoagulants following an acute myocardial infarction.

The observational and randomized studies differed with respect to several failure rates (see Table 3). For some treatment comparisons there were dissimilar failure rates between the types of studies for both treatment and control groups (TENS for postoperative pain and early discharge following childbirth), and for other treatment comparisons there were significant differences between study designs with respect to the rates for patients with the new treatments, but not for patients on the older treatments (quinidine for the treatment of atrial fibrillation and colposuspension versus two older treatments for urinary incontinence).

**Table 3:** Outcome Differences Between RCTs and OSs

Brief Title (outcome)	Number of Studies	Average Failure Rate (Number of Patients)	
		
		Control**	Treatment
Anticoagulants (MI) [13, 14, 64]			
RCT	6	17% (1748)	14% (2106)
OS	9	31%^† ^(3615)	16%^† ^(2598)
Quinidine (Afib) [13, 14, 28] 3 months			
RCT	6	54% (390)	36% (413)
OS	5	61%* (200)	53% (342)
Trial of labor (Breech) [13, 65]			
RCT	2	2% (128)	3% (182)
OS	6	5% (1043)	4%^† ^(1552)
Colposuspension 1 (Incontinence) [13, 66]			
RCT	2	33% (134)	12% (139)
OS	6	37%^† ^(508)	26% (374)
Colposuspension 2 (Incontinence) [13, 66]			
RCT	2	31% (132)	12% (139)
OS	4	32%^† ^(190)	23%^† ^(349)
TENS (Pain) [13, 14, 67]			
RCT	2	18% (34)	3% (34)
OS	4	76%^† ^(172)	56%^† ^(136)
Early Discharge (Childbirth) [13, 68]			
RCT	1	8% (38)	5% (93)
OS	4	21%^† ^(379)	19%^† ^(402)
Hip Screw (Hip Fx) [13, 69]			
RCT	1	50% (26)	12% (33)
OS	5	35%^† ^(290)	8% (560)
Local Anesthesia (CEA) [11, 13, 70]			
RCT	3	5% (79)	5% (75)
OS	11	5%* (1509)	2% (1713)
HSG (Infertility) [11, 13, 71]			
RCT	5	81% (527)	69% (302)
OS	6	74%^† ^(734)	58%^† ^(1072)

RCT = Randomized Controlled Trial; OS = Observational Study

MI = Myocardial Infarction

Afib = Atrial fibrillation

TENS = Transcutaneous electrical nerve stimulation

Hip Fx = Hip Fracture

CEA = Carotid Endarterectomy

HSG = Hysterosalpingography

** The treatment group is listed in the row title. As seen in Table 1, the control (i.e. comparison) groups are the negative of the listed treatment except for the following: Control group for colposuspension 1 is colporrhaphy, colposuspension 2 is needle suspension, hip fracture is fixed nail plates, CEA is general anesthesia, infertility is water soluble medium.

Significance testing was only done to test heterogeneity among the failure rates for the observational studies

* P < .05 for test of heterogeneity of failure rates combined to create the average

† P < .001 for test of heterogeneity of failure rates combined to create the average

As indicated in Table 3, several studies show considerable heterogeneity in results among OSs. For each treatment comparison there was statistically significant variation in failure rates for at least one of the treatments. There was statistically significant heterogeneity in the odds ratios for studies of anticoagulants, colporrhaphy, needle suspension, and hysterosalpingography. Despite small numbers of studies in each treatment area, failure rates were significantly correlated for studies of anticoagulants (r = 0.79, p = 0.01), trial of labor (r = 0.75, p = 0.08), early discharge (r = 0.99, p = 0.01), hip screws (r = 0.93, p = 0.02), and local anesthesia (r = 0.66, p = 0.03). This correlation might be explained by substantial differences among study features that influence failure rates.

### Reporting of treatments and outcomes in OSs

Reporting details of the primary treatment, ancillary treatments, and management of intermediate outcomes was uniformly poor. Most aspects of outcome were also poorly reported. However, outcome definitions were generally well reported. Even here there were exceptions: one study of surgical treatment for incontinence defined the subjective outcome only as "cured" [33], and another defined it as "symptom free" [34].

Length of follow-up, which may substantially influence outcome and comparisons of treatments, was usually not well reported. Of the studies reviewed, only two studies of hysterosalpingography and five of surgical treatment for stress incontinence provided both the mean (or median) and range (or other measure of spread) of follow-up times. Eleven studies provided no follow-up information, and the remainder provided only one number (median, minimum, or undefined).

### Considerations of patient selection in OSs

Even though the majority of studies were based on chart abstraction, none described methods for reducing selection or information bias.

Results from studies were sometimes combined, even though they differed with respect to potentially important patient characteristics. For example, studies of surgical treatment for incontinence varied with respect to exclusions due to previous surgery for incontinence, detrussor instability, and other pathologic findings. Another example is that criteria for studies of local anesthesia for carotid endarterectomy varied on the basis of whether patients were included who were simultaneously undergoing a coronary artery bypass grafting procedure or who had an acute stroke. Among studies of early discharge, one unique inclusion criteria was caesarian delivery [35] and another was primiparity [36]. Of the two studies of HSG that provided detail on inclusion and exclusion criteria, one required infertility for at least two years [37] and a second required infertility for only one year [38].

In Table 4 articles are rated for their reporting of patient characteristics in a descriptive table. Articles were rated as 'A' if they reported at least one item in each of the categories of medical history, demographics, and clinical assessment. Even with these minimal criteria a minority of studies were categorized as 'A'; the only treatment areas that had primarily 'A's were local anesthesia for carotid endarterectomy and colporrhaphy or needle suspension for incontinence. For one treatment area, early versus conventional discharge, none of the OSs provided information on maternal comorbidities or other relevant aspects of medical history.

**Table 4:** Reporting of Patient Characteristics and Efforts to Assess and Control Confounding

	Quinidine (Afib) (n = 6)	Trial of Labor (Breech) (n = 6)	Colposuspension 1 (Incontinence) (n = 6)	Colposuspension 2 (Incontinence) (n = 4)	Early Discharge (Childbirth) (n = 4)	Hip Screw (Hip Fx) (n = 5)	Local Anesthesia (CEA) (n = 11)	HSG (Infertility) (n = 6)
Patient Characterization*								
	A = 4	A = 3	A = 5	A = 3	A = 0	A = 3	A = 7	A = 1
	B = 2	B = 3	B = 1	B = 1	B = 3	B = 1	B = 4	B = 3
	C = 0	C = 0	C = 0	C = 0	C = 1	C = 1	C = 0	C = 0
	D = 0	D = 0	D = 0	D = 0	D = 0	D = 0	D = 0	D = 2
Treatment Selection↖								
	A = 0	A = 0	A = 0	A = 0	A = 1	A = 1	A = 5	A = 3
	B = 0	B = 0	B = 0	B = 0	B = 1	B = 0	B = 3	B = 0
	C = 0	C = 6	C = 5	C = 4	C = 2	C = 0	C = 1	C = 1
	D = 6	D = 0	D = 1	D = 0	D = 0	D = 4	D = 2	D = 2
Comparison of risk factors*								
	A = 0	A = 2	A = 5	A = 3	A = 0	A = 1	A = 5	A = 1
	B = 3	B = 3	B = 1	B = 0	B = 3	B = 2	B = 6	B = 2
	C = 0	C = 1	C = 0	C = 0	C = 1	C = 0	C = 0	C = 0
	D = 3	D = 0	D = 0	D = 1	D = 0	D = 2	D = 0	D = 3
Statistical Adjustment‡								
A	A = 0	A = 0	A = 0	A = 0	A = 1	A = 0	A = 1	A = 0
B	B = 1	B = 3	B = 1	B = 2	B = 0	B = 2	B = 0	B = 0
C	C = 5	C = 3	C = 5	C = 2	C = 3	C = 3	C = 10	C = 6

Afib = Atrial fibrillation

Hip Fx = Hip Fracture

CEA = Carotid Endarterectomy

HSG = Hysterosalpingography

* Characterizations or comparisons were given an 'A' if they included least one element in each of the following categories: demographics, medical history, and clinical assessment. They were given a 'B' if they included one medical history or clinical assessment variable, a 'C' if they included one demographic variable, and a 'D' if there were no characterization or comparisons.

↖ Treatment selection methods were given an 'A' if they probably reduced confounding, a 'B' if the effect on confounding was uncertain, a 'C' if confounding was probably increased, and a 'D' if they were not described.

† Statistical adjustment was given an 'A' for using multiple regression, 'B' for using stratification, and 'C' for no adjustment.

### Factors that influence confounding

Table 4 also describes how study characteristics were reported that could influence confounding. Confounding was more likely in two studies because subjects on one treatment were treated several years previously compared with subjects on another. Confounding was also more likely in other studies (the majority of trials of labor and surgery for incontinence and half the studies of early discharge [32,33]) because treatment was allocated on the basis of patient characteristics likely to influence the possibility of treatment failure. Confounding may have been less likely if the physicians treating the patients used only one procedure. This occurred in a few studies of local anesthesia for carotid endarterectomy, hip screws for hip fracture, and contrast media for HSG. Confounding was considered less likely in another study because of an abrupt change in patient care [35]. In several studies it was not possible to assess how patient preferences may have influenced confounding [39-42].

Table 4 shows whether studies assessed the possibility of confounding by comparing patients on the two treatments with respect to at least one variable from the categories of medical history, demographics, and clinical assessment. Most studies did not make these comparisons; the few that did should have evaluated additional potential confounders. In addition, once potential confounders were identified, the studies made only minimal use of statistical methods to control for confounding. A few studies attempted to control for confounding by stratifying on the basis of some risk factors, but only one study performed a regression analysis that adjusted for multiple risk factors [43].

### Reasons for OS heterogeneity

We found evidence that variation in outcome definition and length of follow-up caused heterogeneity in results. For example, in studies of trial of labor, the study with the lowest failure rate [44] was also the study that defined poor outcome in the newborn as a five-minute Apgar score less than five, instead of less than seven as used in other studies (the lower the score the more likely the newborn is to require resuscitation). For studies of early discharge, the lowest failure rate came from a study that examined post-operative complications of C-section patients, and the highest rates came from a study that included many common symptoms in the definition of maternal morbidity (e.g., cold, flu, and constipation). In the studies comparing colposuspension to either needle suspension or colporrhaphy, the lowest failure rates in the colposuspension groups came from studies in which follow-up was less than one-year, and those low failure rates were very similar to the RCTs, both of which had follow-up of one year. For the study of HSG with the lowest odds ratio (0.98) [37], the duration of follow-up was two years, as compared to the other studies which had follow-up of one year or less. It is possible that infertility problems that improved with oil-contrast media may have resolved in any case over a two-year period.

### Reasons for differences between observational and randomized studies

Differences for the studies of TENS for postoperative pain and early discharge following childbirth may have been due in part to dissimilar definitions of failure. The OSs of TENS defined this as whether or not a patient received post-operative medications. The randomized controlled trials used verbal ratings of pain that were dichotomized into "satisfactory" or "unsatisfactory". For studies of early discharge, the randomized controlled trial defined failure as maternal problems requiring physician referral. These problems were primarily infections: urinary tract infections, episiotomy infection, mastitis, subinvolution, and endometritis. Most OSs defined failure as maternal problems determined from physical assessment or self-report. Since these problems included constipation, flu-like symptoms, and lethargy as well as infections, failure rates were generally higher for observational than for randomized studies. The one exception was an observational study that examined outcomes post-caesarian section and defined failure as fever, wound infection etc [35]. The failure rates for this study were 6% for early discharge and 7% for the conventional group, which are similar to the rates from randomized studies. Without this study of C-section patients, the overall failure rates for the observational and randomized studies would have differed even more.

The primary concern about OSs is confounding. There was evidence of obvious confounding that was not taken into account in three treatment comparisons: 1) influence of anticoagulants on survival of myocardial infarction (historical controls treated several years earlier [45-47] and anticoagulants preferentially given to younger patients and patients at lower risk for other reasons [48,49]) 2) quinidine for the treatment of arrhythmias (significantly higher [50,51] rates of valvular heart disease in the quinidine group), and 3) colposuspension versus anterior colporrhaphy (substantially and significantly higher rates in the colposuspension group of severe pre-surgery incontinence [52]). [32,34] In no study showing obvious confounding did the authors assess or adjust for confounding, or even raise it as a concern.

## Discussion

Previous studies have compared results of OSs and RCTs. The present investigation was the first to evaluate what design features could have influenced results of OSs and, therefore, the comparisons of results from OSs and RCTs. We found evidence that some factors unrelated to validity (treatment specifics, patient characteristics, and methods of measuring outcomes) could have influenced results in some of the studies. However, the comparisons of RCTs and OSs (and in many cases the original meta-analyses that combined the studies) did not take these study features into account. Therefore, it is possible that some differences between some RCTs and OSs may be due to factors other than lack of validity of OSs.

Clearly, however, a critical validity issue (confounding) influenced the results of some OSs. Patients on some treatments differed substantially from patients on another with respect to risk factors or ancillary treatments that probably influenced outcomes and altered the observed relative effectiveness of the two treatments. Unfortunately, few studies assessed the possibility of confounding, and almost none made a sophisticated effort to control for it. Because of the potential for confounding to invalidate the results of OSs, the lack of concern with confounding was surprising and disturbing.

The primary finding of this investigation was that few OSs of medical treatments provided sufficient information for their results to be adequately interpreted. The poor reporting impaired the ability of the systematic reviews and meta-analyses that included these articles to explain differences in results or decide how results should be combined. It may also have contributed to our inability to account for most of the variation in results among OSs and the causes of discrepancies between OSs and RCTs. Differences may have occurred because the OSs and RCTs evaluated different treatments, defined outcomes differently, or had obvious confounding. The OSs reviewed did not provide sufficient evidence to assess whether they were invalid because of undetectable and unavoidable confounding. This type of confounding is of greatest concern in OSs and may have been responsible for differences between OSs and RCTs of hormone replacement therapy. Undetectable confounding may be less likely when patients have little influence on treatment choice, such as decisions about a specific surgical procedure.

In summary, our study provided little evidence either for or against the validity of OSs. However, it suggested that causes of differences previously found between OSs and RCTs are difficult to determine. The OSs we examined may not be representative of all OSs that evaluated medical treatments. However, the severe reporting problems in the 61 studies reviewed suggest that many other published studies provide inadequate information. Without improved standards for reporting, it will be difficult to assess how OSs on a given topic should be interpreted or, more generally, the appropriate role for OSs in the evaluation of medical treatments. Standards can be improved by developing criteria for studies and involving more researchers with a strong epidemiological background in the design, reporting, and review of OSs of medical treatments.

## Competing interests

The author(s) declare that they have no competing interests.

## Authors' contributions

AH was responsible for much of the study design and writing. SB, MC, YB, and KB reviewed the articles, helped develop the format for abstraction, and examined relationships between study characteristics and results. DL and MS helped with the conceptualization and writing of the article.
